# Death from the New Anæsthetic

**Published:** 1873-11-01

**Authors:** 


Death from the New Anæsthetic.—Dr. Richardson, of England, the indefatigable searcher after new anæsthetics, has recently introduced into that country the use of a mixture of sulphuric ether and bi-chloride of methyl, hoping to gain thereby the full safety of ether, with some of the promptness of effect characterizing the methyl.

The mixture was called "methylene ether," and began to be a great favorite in some hospitals. It was particularly convenient, because a patient could be fully brought under its influence in less than two minutes. The bright hopes based on its use, however, have been dimmed, for the last number of an English medical journal brings us an unmistakable death produced by its use already. A vigorous patient inhaling it died on the table, before the operation was commenced.

